# Neurobiological mechanisms involved in maternal deprivation-induced behaviours relevant to psychiatric disorders

**DOI:** 10.3389/fnmol.2023.1099284

**Published:** 2023-04-13

**Authors:** Natália Cristina Zanta, Nadyme Assad, Deborah Suchecki

**Affiliations:** Departamento de Psicobiologia, Escola Paulista de Medicina, Universidade Federal de São Paulo, São Paulo, Brazil

**Keywords:** early life stress, maternal deprivation, HPA axis, neurotransmitters, neuropeptides, affective behaviour

## Abstract

Parental care is essential for proper development of stress response and emotion-related behaviours. Epidemiological studies show that parental loss in childhood represents a major risk factor for the development of mental disorders throughout the lifespan, including schizophrenia, depression, and anxiety. In most mammalian species, the mother is the main source of care and maternal behaviours regulate several physiological systems. Maternal deprivation (DEP) for 24 h is a paradigm widely used to disinhibit the hypothalamic–pituitary–adrenal axis response to stress during the stress hyporesponsive period. In this mini-review we will highlight the main DEP-induced neurobiological and behavioural outcomes, including alterations on stress-related hormones, neurogenesis, neurotransmitter/neuromodulatory systems and neuroinflammation. These neurobiological changes may be reflected by aberrant behaviours, which are relevant to the study of mental disorders. The evidence indicates that DEP consequences depend on the sex, the age when the DEP takes place and the age when the animals are evaluated, reflecting dynamic plasticity and individual variability. Individual variability and sex differences have a great relevance for the study of biological factors of stress resilience and vulnerability and the DEP paradigm is a suitable model for evaluation of phenotypes of stress- and emotion-related psychopathologies.

## 1. Introduction

Disruption of parent-infant relationship, including parental loss, abandonment or incarceration, represent risk factors for the development of depression (Berg et al., 2016), schizophrenia (Agid et al., 1999), and posttraumatic stress disorder (PTSD) in adulthood (Koenen et al., 2007; Lis-Turlejska et al., 2008). These events lead to dysregulation of the hypothalamic–pituitary–adrenal (HPA) axis stress response (Luecken, 1998; Tyrka et al., 2008a,b; Hagan et al., 2011; Kaplow et al., 2013), which may impact the trajectory of neurodevelopment and functioning of several brain systems. Maternal deprivation for 24 h (DEP) was developed in rats and mice to understand the effects of mother-infant disruptions on neurodevelopment, physiology and behaviour (Levine et al., 1991; Suchecki, 2018). It is a model of extreme neglect (Oomen et al., 2010) and transient loss of parental care (Suchecki, 2018). Maternal separation and DEP have been used as synonyms, but there are important differences between these protocols as well as in the outcomes and physiological mechanisms involved (for a comprehensive review on the subject, see Faturi et al. (2010)).

## 2. DEP effects on the HPA axis

In rats and mice, the importance of maternal presence goes beyond nourishment, induction of excretion and thermoregulation, as specific behaviours can stimulate physiological processes in the pups, such as heart rate, motor activity, sleep–wake cycle (Hofer, 1994; Hofer, 2006) and release of growth hormone (Evoniuk et al., 1979) or inhibit others, like the adrenocortical stress response (Levine et al., 1991). The stress hyporesponsive period (SHRP) is a hallmark of the ontogenesis of the stress response in rats and mice. It is characterised by adrenal insensitivity to its trophic hormone, adrenocorticotropin (ACTH) (Witek-Janusek, 1988) and lower responsiveness to stressful stimuli (Witek-Janusek, 1988; Walker et al., 1991), resulting in low and stable corticosterone (CORT) levels. In mice this period spans from days 1 to 12, and in rats, from days 4 to 14 (reviewed in van Bodegom et al. (2017)). Specific maternal behaviours regulate the stress response, such that nursing inhibits CORT and anogenital licking suppresses the ACTH stress response (Stanton et al., 1987; Rosenfeld et al., 1993; Suchecki et al., 1993; van Oers et al., 1998). Therefore, in both rats and mice, DEP during the SHRP augments the stress response (Levine et al., 1991; Schmidt et al., 2004; Enthoven et al., 2010; Faturi et al., 2010). The elevation of CORT basal and stress-induced levels is observed from 8 h of separation, reaching the highest levels by 24 h in both species (Levine et al., 1991; Schmidt et al., 2004). The effects of three 8 h separations on CORT response do not accumulate, since reunion with the mother resets the system to control (CTL) levels (Rosenfeld et al., 1992). After DEP, the CORT and ACTH responses to a saline injection remain elevated for at least 2 h (Suchecki et al., 1995; Girardi et al., 2014).

In rats, corticotropin releasing hormone (CRH) mRNA expression in the paraventricular nucleus of the hypothalamus (PVN) reaches adult levels by PND 4 (Baram and Lerner, 1991) and DEP on PND 9 (DEP9) reduces CRH levels in the median eminence (Pihoker et al., 1993), suggesting that DEP increases the release of this neuropeptide. In mice, CRH mRNA expression is high between PNDs 1 and 9, decreasing between PNDs 12 and 16 (Schmidt et al., 2003). In rats, DEP on day 9 (Schmidt et al., 2002) or on day 11 (DEP11) reduces CRH mRNA expression (Smith et al., 1997), whilst it enhances ACTH and CORT levels, suggesting that maternal inhibition of the HPA axis is confined to the peripheral components, e.g., pituitary and adrenals. Moreover, in 15 day-old mice, gene expression for CRH, vasopressin, glucocorticoid and mineralocorticoid receptors was similar between DEP9 and CTL animals (Reshetnikov et al., 2020).

Besides its immediate effects, DEP increases stress-induced CORT levels and adrenal relative weight in DEP3 male and female adolescent rats (Miragaia et al., 2018; Ceschim et al., 2021). CORT stress response is also higher in adult, but not aged, DEP4, DEP9 and DEP18 rats (Lehmann et al., 2002). DEP11 blunts the ACTH (van Oers et al., 1998) and CORT responses to a saline injection (Suchecki and Tufik, 1997) in juvenile rats (20–30 day olds), as well as the ACTH response to a hypertonic saline injection in adult DEP11 male and female rats (Faturi et al., 2010). However, in response to a session in the elevated plus maze, which represents a psychological stress, DEP11 adults of both sexes display a CORT response similar to their CTL counterparts (Cabbia et al., 2018). These results indicate that DEP induces plastic changes in the peripheral parts of the HPA axis that are not permanent and may depend on the nature of stressful stimulus (physical vs. psychological).

The immediate disinhibition of the HPA axis by DEP is a consistent finding, but very little is known about how other systems are altered right after this adversity. The long-term outcomes on brain systems and behaviour are far more complex and seem to be dependent on the sex, age of DEP and age at evaluation. In this **mini review** we will briefly describe the main outcomes of DEP on PND 3 (DEP3), 9 (DEP9) or 11 (DEP11), on several neurobiological systems and, when possible, we will highlight the relationship between changes in neurobiology and behaviours relevant to psychiatric disorders. These ages were chosen because they result in behavioural changes relevant for the study of co-morbid anxiety and depression (DEP3), depression (DEP9 and DEP11) and schizophrenia, and depression (DEP9).

## 3. DEP consequences on neurobiology and affective behaviour

**Figure 1:**
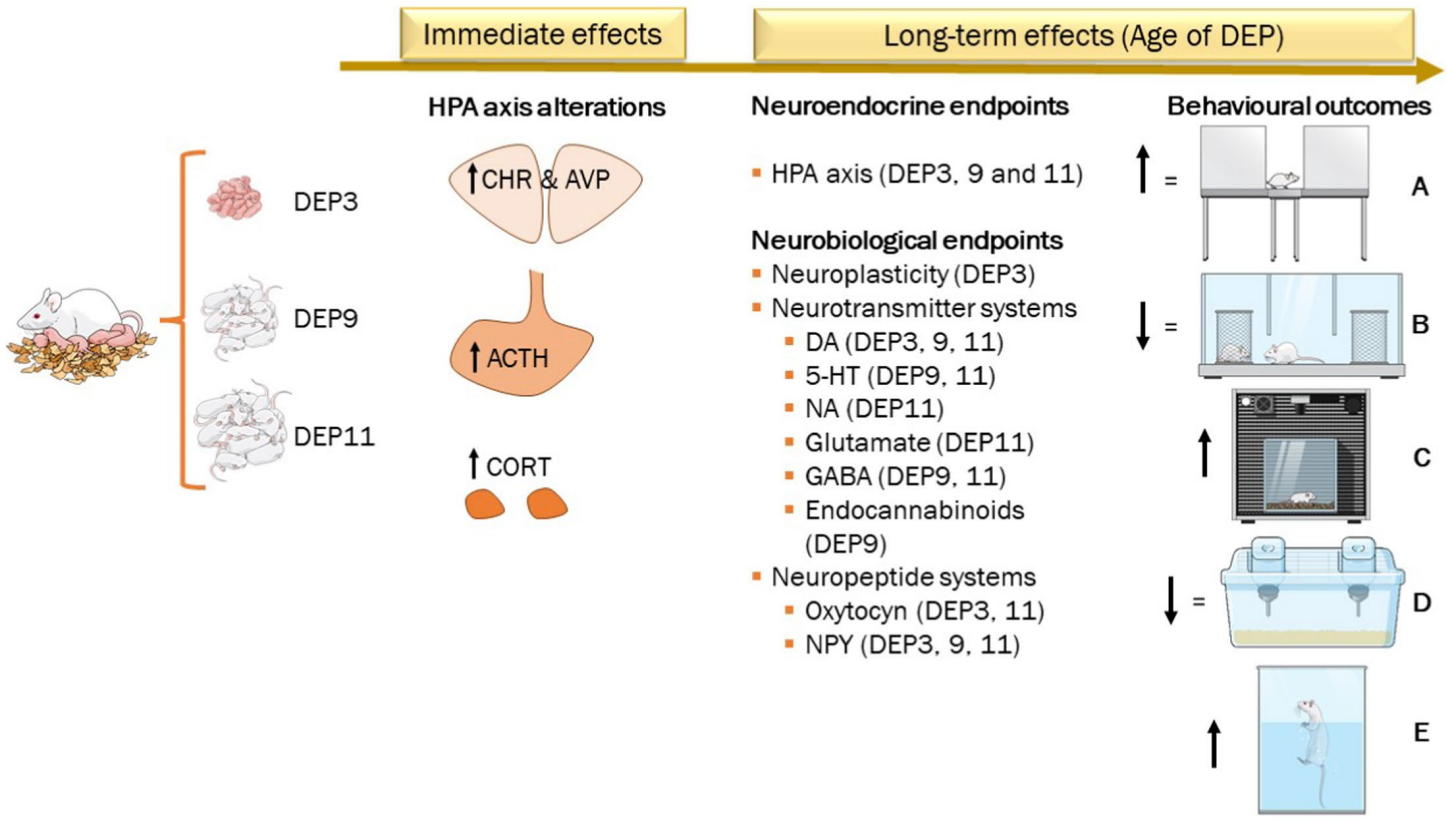
Immediate effects of maternal deprivation (DEP) on postnatal days (PND) 3 (DEP3), 9 (DEP9) or 11 (DEP11) on the hypothalamic–pituitary–adrenal (HPA) axis and the long-term effects neurobiological and behavioural endpoints. Although DEP increases corticosterone (CORT) levels, the magnitude of this effect depends on the age. **(A)** Anxiety-like behaviour measured in the elevated plus maze, **(B)** Social investigation test, **(C)** Prepulse inhibition, **(D)** Sucrose preference tests, and **(E)** Immobility in the forced swim test. Images were obtained from Mind the Graph (www.mindthegraph.com).

Table 1 presents the main results of the studies included in this mini review. Figure 1 shows a graphical abstract of the neurobiological and behavioural parameters assessed in the studies that employed DEP on postnatal days 3, 9, and 11.

**Table 1:** Effects of maternal deprivation on different neurobiological systems and behaviours.

Long-term outcomes		Age of deprivation	Sex		References
			Male	Female	
Neurogenesis	DCX-IR	PND 3	↑	↓	Oomen et al (2009), Oomen et al. (2010), Oomen et al. (2011)
	BDNF cell proliferation cell death	PND 3 PND 9 PND 11	↓ PFC, HPC ↑ DG ↓ DG ↑ DG, parietal cortex, cerebellar cortex	↓ PFC, HPC	Marco et al. (2013), Reshetnikov et al. (2020), Fabricius et al. (2008), Zhang et al. (2002)
Neurotransmitters	TH-mRNA levels	PND 3	↑	–	Rots et al. (1996)
	DA neurons	PND 9	↑ *substancia nigra*	↑ *substancia nigra*	Kapor et al. (2020)
	D2 receptor	PND 9	↑ *striatum* ↓ PFC	↑ *striatum* ↓ PFC	Rentesi et al. (2013)
	DA levels	PND 11	↑ FC	↑ AMY	Cabbia et al. (2018)
	5-HT levels	PND 9	↑ HPC, *striatum*	↑ HPC	Llorente et al. (2010)
	5-HT turnover	PND 9	↑ HPT	–	Llorente et al. (2010)
	5-HT1A receptors	PND 9	↓ HPC	–	Henn et al. (2021)
	5-HT2A receptors	PND 9	↓ *striatum*, PFC ↑ AMY	–	Rentesi et al. (2013)
	5-HT levels	PND 11	↓ FC ↑ HPT	–	Cabbia et al. (2018)
	NA levels	PND 11	↓ HPC	↑ FC ↓ HPC	Barbosa Neto et al. (2012), Cabbia et al. (2018)
	NMDA receptor	PND 9	↓ HPC, PFC	↑ CP	Roceri et al. (2002), Zamberletti et al. (2012)
	Glutamate levels	PND 11	↑ HPC	–	Barbosa Neto et al. (2012), Janetsian-Fritz et al. (2018)
	GAD-IR	PND 9	↓ cortex		Janetsian-Fritz et al. (2018), Poleksic et al. (2021)
	GABA levels	PND 11	–	↓ HPC	Barbosa Neto et al. (2012)
	CB1 receptor expression	PND 9	↓ HPC ↑ PFC	↓ HPC ↑ PFC	Suarez et al. (2009), Marco et al. (2014)
	CB2 receptor expression	PND 9	↑ HPC	↑ PFC	Suarez et al. (2009), Marco et al. (2014)
Neuropeptides	OT-ir cells	PND 3	–	↑ SON, PVN	Ceschim et al. (2021)
	NPY-ir cells	PND 3 and 11	↓ ARC, BLA, dHPC	↓ ARC, BLA, dHPC	Wertheimer et al. (2016), Miragaia et al. (2018)
	NPY levels	PND 9	↓ HPC	–	Husum et al. (2002)
Neuroinflammation	Iba-1-IR GFAP-IR	PND 9 PND 9	↑ PFC (at PND 10) No differences at PND 60 ↑ CA1, CA3, DG (PND 13) ↑ HPC	↑ DG (PND 13)	Viveros et al. (2007), Kaur et al. (2017), Marco et al. (2013)
Behaviour	Schizophrenic-like behaviour	PND 3 PND 9 PND 9	↓ PPI (effect was smaller than on PND 9) ↓ PPI ↓ PPI	— ↓ PPI —	Ellenbroek et al. (1998), Husum et al. (2002)
	Anxiety-like behaviour (avoidance of open arms in the EPM)	PND 3	↑	↑	Miragaia et al. (2018)
		PDN 11	No change (Adolescence) ↑ (Adults)	No change (Adolescence) ↑ (Adults)	Miragaia et al. (2018), Barbosa Neto et al. (2012)
	Depressive-like behaviour (floating in FST)	PND 3	↑	↑	Miragaia et al. (2018)
		PND 9	↓ (Early adolescence)	↓ (Early adolescence)	Llorente et al. (2007)
		PND 11	↑	↑	Miragaia et al. (2018)

AMY, amygdala; ARC, arcuate nucleus of the hypothalamus; BDNF, brain derived neurotrophic factor; BLA, basolateral amygdala; CA3/CA1, cornu ammonis of HPC; DA, Dopamine; DCX-IR, doublecortin immunoreactivity; DG, dentate gyrus; dHPC, dorsal hippocampus; EPM, elevated plus maze; FC, frontal cortex; FST, forced swim test; GFAP, glial fibrillary acidic protein; HPC, hippocampus; HPT, hypothalamus; Iba-1, ionized calcium-binding adapter molecule 1; IR, immunoreactivity; NA, noradrenaline; PFC, prefrontal cortex; PPI, prepulse inhibition; PVN, paraventricular nucleus of the hypothalamus; SON, supra-optic nucleus of the hypothalamus.

### 3.1. Neurogenesis and neuroplasticity

Neurogenesis depends on several factors, including brain derived neurotrophic factor (BDNF). Neurogenesis rate peaks between the last prenatal week and the first two weeks after birth (Morgane et al., 2002). Most granule hippocampal neurons are produced during the SHRP (Schlessinger et al., 1975). CORT, on the other hand, impairs BDNF production and neurogenesis (Tanapat et al., 1998; Schoenfeld and Gould, 2012). Therefore, DEP-induced persistent elevations in CORT levels during this period could be harmful for adequate hippocampal development. One study confirmed this prediction as 12 day old rats submitted to DEP11 exhibited more cell death, not only in the dentate gyrus (DG), but also in the parietal and cerebellar cortices. Moreover, after DEP11 there was more cell death in white matter tracts and this effect was proportional to the increase in nerve growth factor mRNA (Zhang et al., 2002). The short-term effects on hippocampal neurogenesis were investigated in 15 day old DEP9 male mice and the results show greater cell proliferation in the DG compared to CTL mice (Reshetnikov et al., 2020). These apparently contradictory findings may result from different ages of DEP and/or from a recovery process that might have taken place in Reshetnikov and colleague’s study (Reshetnikov et al., 2020).

Studies using DEP3 show a sex-dependent outcome, with increased cell proliferation and DCX-immunoreactivity (IR) in 21-day old male rats and reduced neurogenesis in female rats (Oomen et al., 2009, 2010, 2011). In line with these results, DEP3 females display more freezing in a cued fear conditioning task, indicating greater efficiency in amygdala-dependent fear memory (Oomen et al., 2011). In males, the neurogenesis inducing effect of DEP was transient: at 10 weeks of age, DEP3 impaired proliferation, survival and differentiation in the caudal part of the hippocampus (HPC), along with impaired spatial learning and improved emotional learning (Oomen et al., 2010). In adult male mice, DEP9 only produces a small decrease in the number of DG neurons (about 20%) (Fabricius et al., 2008). These findings reinforce the need for longitudinal evaluations to better understand the plastic changes induced by DEP.

In adolescent rats, DEP9 decreases the production of BDNF and PSD95, a marker of postsynaptic density, in the prefrontal cortex (PFC) and HPC of male and female rats (Marco et al., 2013). In addition, DEP9 reduces the levels of synaptophysin in PFC and neural cell adhesion molecule in HPC of males and females. Despite the similar neurobiological effects on both sexes, only DEP9 females display impairment in the novel object test, a hippocampal-dependent task (Marco et al., 2013).

### 3.2. Neurotransmitter systems

Several studies explored the effects of DEP on the activity of neurotransmitter systems. DEP3 induces hyperactivity of the dopaminergic system, with higher tyrosine hydroxylase mRNA levels in the substantia nigra of adolescent rats and greater sensitivity of DA-type 2 (D2) receptor to apomorphine than CTL rats (Rots et al., 1996). DEP9 increases the volume and number of dopaminergic neurons in the substantia nigra, pars compacta and reticulata, and in the ventral tegmental area of adolescent and adult rats (Kapor et al., 2020). Despite that, another study (Rentesi et al., 2013) showed that DA levels and turnover in the striatum were lower in DEP9 and that D2 receptor protein levels were higher in the striatum and lower in the PFC (Rentesi et al., 2013). DEP11 rats also exhibit high DA levels in the frontal cortex of adult males and in the amygdala of females (Cabbia et al., 2018).

DEP9 is a translational model of schizophrenia in adult male and female rats, who show impaired prepulse inhibition (PPI), a behaviour related to sensory-motor gating, an outcome that was not observed in DEP3 male rats (Ellenbroek et al., 1998). Moreover, treatment with haloperidol or quetiapine reversed the effects of DEP9 on PPI, fulfilling predictive validation of animal models (Ellenbroek et al., 1998). Collectively, these data indicate that DEP at different ages results in hyperactivity of the dopaminergic pathways and, depending on the age, the behavioural outcomes are relevant to the study of schizophrenia.

Serotonin (5-HT) and noradrenaline (NA) systems are altered by DEP in an anatomical-and sex-dependent manner. DEP9 adolescent male rats show higher 5-HT levels in the HPC and striatum than CTL counterparts, whilst DEP9 females exhibit higher 5-HT hippocampal levels than CTL ones. Moreover, 5-HT turnover in the hypothalamus (HPT) of DEP9 adolescent males is higher than that of CTL rats (Llorente et al., 2010). In line with the increased 5-HT hippocampal levels, lower 5-HT1A receptor protein levels were found in DEP9 adolescent male rats (Henn et al., 2021). In adult DEP9 male rats, 5-HT2A receptors are reduced in the striatum and PFC and increased in the amygdala (AMY) (Rentesi et al., 2013). Compared to CTL rats, DEP11 adult males have lower 5-HT levels in the FC and higher in the hypothalamus (HPT) (Cabbia et al., 2018). Furthermore, DEP11 males display more hypoxia-induced panic-like behaviour than CTL rats and depletion of 5-HT levels by parachlorophenylalanine treatment increases panic-like behaviour in CTL, but has no effect in DEP11 rats, suggesting that 5-HT activity is already impaired in these animals (Rosa et al., 2022). DEP11 female rats display higher NA levels in the FC (Cabbia et al., 2018) and lower levels in the HPC (Barbosa Neto et al., 2012) than their CTL counterparts. Thus, DEP alters the activity of 5-HT system in an age-dependent manner: DEP9 increases and DEP11 decreases this neurotransmitter levels. DEP11 (the only age evaluated) causes small and sex-specific changes in the NA system, with no impact on emotional behaviour.

Few studies explored the effects of DEP on amino acid neurotransmitter systems. DEP9 reduces the expression of NR-2A and NR-2B subunits of the NMDA glutamatergic receptor in the HPC and PFC of adult male rats (Roceri et al., 2002) and increases binding of this receptor in the caudate-putamen of adult female rats (Zamberletti et al., 2012). Protein levels or glutamic acid decarboxylase (GAD 67)-IR, one of the GABA synthesizing enzymes, are lower in many cortical areas of DEP9 compared to CTL male rats (Janetsian-Fritz et al., 2018; Poleksic et al., 2021). DEP11 alters hippocampal levels of amino acid neurotransmitters in a sex-dependent manner. Adult male rats exhibit higher levels of the excitatory amino acids glutamate and aspartate and lower levels of the inhibitory amino acid taurine, whilst females show a slight reduction of taurine and a major reduction in GABA levels. These alterations lead to an imbalance of excitatory/inhibitory activity in the HPC of DEP11 compared to CTL rats (Barbosa Neto et al., 2012), which may compromise the adequate functioning of this brain structure.

The endocannabinoid (EC) system is a buffering regulator of the stress response (Viveros et al., 2007) and only a few studies explored the effects of DEP9 on this system. The findings show that DEP9 reduces cannabinoid receptor type 1 IR (CB1-IR) in male pups and increases CB2 receptor-IR in both sexes in several hippocampal fields of 13-day old pups (Suarez et al., 2009). In a subsequent study, the same group performed a thorough investigation of DEP9 effects on gene expression of CB1, CB2, transient receptor potential vaniloid-and G-coupled receptors and all enzymes involved in the biosynthesis and degradation of EC in several brain regions. All parameters were increased in the PFC of DEP9 males and HPC of DEP9 females (Suarez et al., 2010; Marco et al., 2014). Acute treatment of CTL males with WIN55,212-2 – a CB1 receptor agonist—reduced exploration of the open arms in the elevated plus maze. However, this effect was absent in DEP9 males. This drug treatment also increased CORT levels irrespective of sex and group, but this effect was more intense in DEP9 adolescent males (Llorente et al., 2007). In the forced swim test (FST) DEP9 adolescent males initiated floating behaviour faster and both males and females floated longer than CTLs (Llorente et al., 2007). These findings indicate that DEP9 leads to long-term region-specific changes in the EC system; however, the direction of the effect seems to depend on the technique used.

### 3.3. Neuropeptides

In addition to classical neurotransmitters, neuromodulatory systems have also been investigated in the context of DEP. Oxytocin (OT) is mostly synthesized in the supraoptic (SON) and paraventricular PVN nuclei in the HPT and has a role in reproduction (e.g., birth, lactation), regulates social and emotional behaviour and stress-related responses (for review, see Onaka and Takayanagi (2021)). The only study that addressed the influence of DEP on the OT system and social behaviour showed a slight increase of OT-IR in the magnocellular part of the SON and PVN of DEP3 adolescent females in comparison with DEP11 counterparts and an increased investigation of the empty container in the social investigation test in DEP3 adolescent males (Ceschim et al., 2021).

The best studied neuropeptide in the context of DEP is Neuropeptide Y (NPY), the most abundant in the brain which is mainly produced in the arcuate nucleus of the HPT (ARC) (Heilig, 2004), but also in the lateral septum, amygdala, HPC and *locus coeruleus*. Anatomical, pharmacological, and behavioural evidence indicates that NPY is associated with stress resilience and anxiolysis (Heilig et al., 1994; Thorsell et al., 2000; Serova et al., 2014; Thorsell and Mathé, 2017).

Adult DEP9 males display lower NPY levels in the HPC than CTL rats, accompanied by impairment of PPI (Husum et al., 2002), suggesting a possible involvement of low NPY levels with schizophrenia-like behaviour. DEP3 and DEP11 male and female adolescent rats have lower NPY-IR in the ARC, which explains their reduced food intake and lower body weight than CTL rats (Wertheimer et al., 2016). They also have less NPY-IR in the basolateral amygdala and dorsal HPC, although in males, the effect on DEP3 males was more intense than in DEP11 (Miragaia et al., 2018). In both studies, NPY-IR was smaller in females than in males, regardless of the group (Wertheimer et al., 2016; Miragaia et al., 2018). In the latter study, emotional behaviours were assessed and DEP3 male and female young adult rats displayed more anxiety-like behaviour in two tests and more immobility in the FST than DEP11 and CTL counterparts (Miragaia et al., 2018), suggesting that DEP3 results in co-occurrent anxiety and depressive-like behaviours. DEP11 male and female rats did not show changes in anxiety-like behaviour, although they also spent more time floating in the FST, but this effect was subtler than that seen in DEP3 adolescents. Interestingly, in the negative sucrose contrast test, a test that measures the loss of rewarding salience of from a high to low palatable sucrose solution, only DEP3 and DEP11 males were affected, still preferring the low sucrose solution to water. This result can be interpreted as a blunted hedonic response, characteristic of depressive patients. These findings indicate that DEP-induced impairment of NPY function may underlie the expression of behaviours relevant to stress-related psychiatric disorders. Indeed, low NPY levels in the cerebrospinal fluid were observed in depressive (Heilig et al., 2004) and PTSD patients (Yehuda et al., 2006; Sah et al., 2009).

### 3.4. Glial cells and neuroinflammation

Neuroinflammation plays an important role in the pathophysiology of stress-related psychiatric disorders (Dantzer et al., 2008) and many studies show that early life stress can alter microglial development and function (for a recent review, see Catale et al. (2020)), but data on the effects of DEP are scarce.

In 10 -or 60-day old rats, levels of Iba-1, a marker of microglia, in the PFC were unchanged by DEP9. However, immunohistochemistry revealed that DEP9 male pups had more Iba-1 labelling than controls in the medial orbital and infralimbic cortices and this effect vanished at day 60 (Poleksic et al., 2021). DEP9 increased the number of glial fibrillar acidic protein positive cells (GFAP+), a marker of astrocytes, in the CA1, CA3 of 13 day-old male pups, and in the molecular layer of the DG in both sexes (Llorente et al., 2009); GFAP levels were still higher in the HPC of DEP9 than in CTL male rats at 40 days of age (Marco et al., 2013). In mice, the effects of DEP9 were compared with those of maternal separation (MS) from days 2 to 14 (3 h/day) on the morphology of glial cells in the PFC and HPC at day 15 of life. DEP9 increased the number of intermediate glia, whilst MS increased ameboid glia (Reshetnikov et al., 2020), suggesting that these early life adversities retard the maturation of glial cells from ameboid to ramified morphology (Kaur et al., 2017).

One study examined the effects of DEP9 on nociceptive response of rats submitted to peripheral nerve injury and on mRNA expression of interleukin 1β (IL-1β), IL-6, and tumour necrosis factor alpha (TNF-α). DEP9 females displayed greater pain response to a non-painful stimulus, and higher TNF-α expression in the HPC than CTL ones. DEP9 reduced IL-6 expression in the PFC of males. These data show sexually dimorphic effects of DEP9 on nociception and suggest that neuroinflammatory alterations underlie these changes (Burke et al., 2013).

## 4. Final considerations

In rats and mice, DEP leads to immediate changes in the physiological response to stress and long-term consequences on stress response and behaviours relevant to psychiatric disorders. DEP is a useful tool to assess the effects of elevated CORT levels in specific hallmarks of neurodevelopment and how this phenomenon can alter the phenotypic trajectory, leading to vulnerability or resilience. DEP at all ages during the first two weeks of life disinhibits the stress response, but investigation of the immediate effects is restricted to DEP9 and the EC system and neuroinflammation. Despite the immediate effects of DEP on CORT levels, DEP3 and DEP9 lead to greater vulnerability to stress-related psychiatric disorders, whilst the behavioural changes of DEP11 seem to be less intense than the earlier ages. Therefore, longitudinal studies on the impact of DEP3 and DEP11 on neurobiology and behaviour in a sex-dimorphic context could help to understand how this adversity influences molecular and cellular mechanisms involved in resilience and vulnerability throughout the individual’s developmental trajectory. Disclosure of these mechanisms can provide the seed to develop new and more effective treatments and even preventive interventions for maternally/paternally deprived children and improve their mental health.

## Author contributions

NZ and NA wrote the first draft of the manuscript and prepared the table. DS edited the manuscript, table, and prepared the figure. All authors contributed to the article and approved the submitted version.

## Funding

The original studies cited in this review were supported by grants from AFIP, CAPES (Financial code 001), FAPESP (grants # 94/0262-2; 2006/06415-4; 2015/26364-4; 2019/21980-0) and CNPq (grants # 400039/1994-0; 470449/2008-0; 302294/2012). NZ is the recipient of a Ph.D. fellowship from CAPES and NA is the recipient of a Ph.D. fellowship from CNPq. DS is the recipient of a Research fellowship from CNPq (grant # 302608/2019-2). Payment for this publication was made possible by CAPES (Financial code 001).

## Conflict of interest

The authors declare that the research was conducted in the absence of any commercial or financial relationships that could be construed as a potential conflict of interest.

## Publisher’s note

All claims expressed in this article are solely those of the authors and do not necessarily represent those of their affiliated organizations, or those of the publisher, the editors and the reviewers. Any product that may be evaluated in this article, or claim that may be made by its manufacturer, is not guaranteed or endorsed by the publisher.
